# Supplementary figures and images for: Absolute measurement of the tissue origins of cell-free DNA in the healthy state and following paracetamol overdose
Source: BMC Med Genomics. 2020 Apr 6;13:60. doi: 10.1186/s12920-020-0705-2 (PMC7133021; doi:10.1186/s12920-020-0705-2)

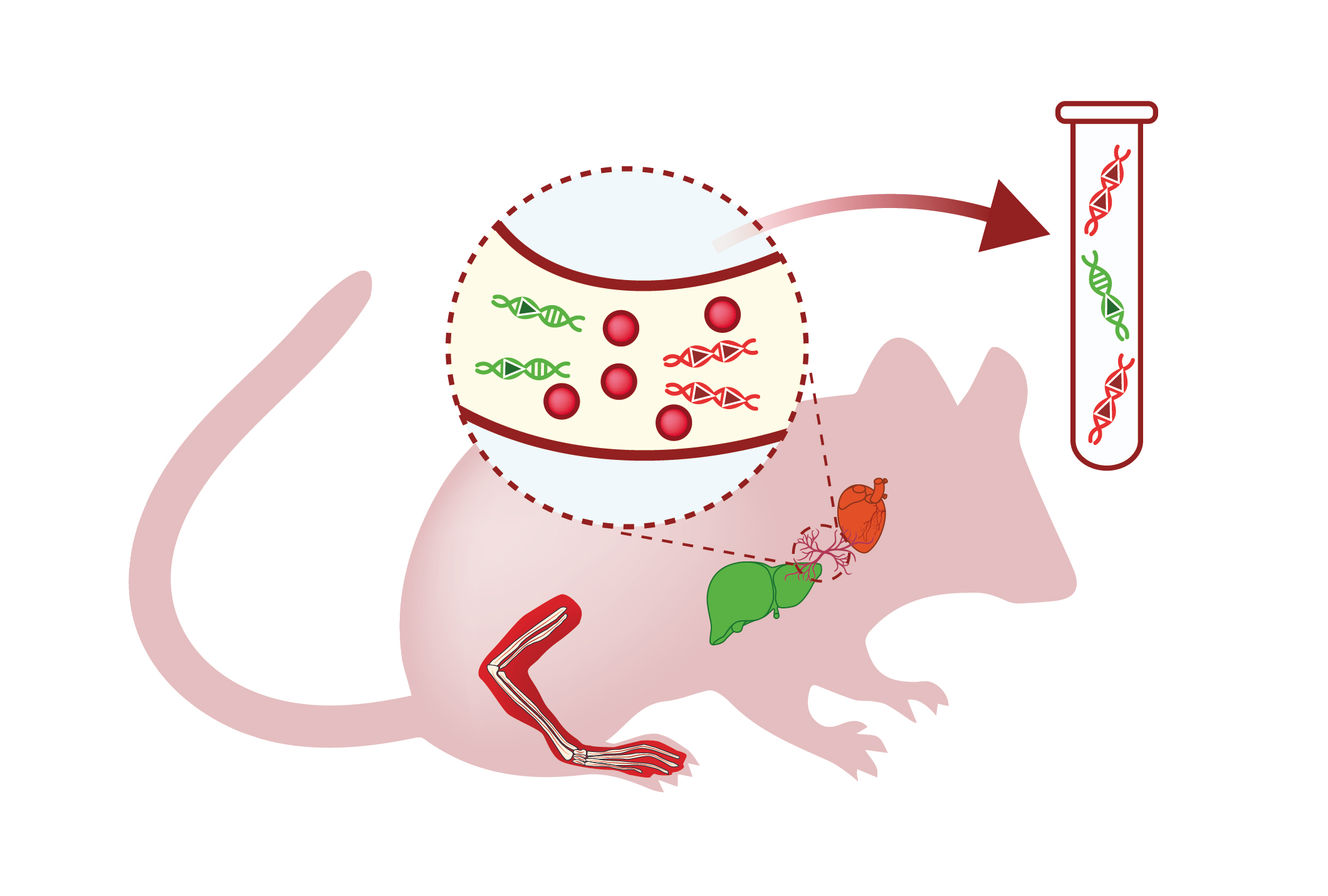

Supplement: Supplementary file 1 — Additional file 1: Figure S1. A schematic representation of the overall strategy for absolute quantitation of the tissue origins of cell-free DNA. Description of data: A conditional knockout is generated in the tissue of interest (e.g. liver) for each mouse model, containing a tissue/cell-specific Cre recombinase and the reporter gene floxed mT/mG. Cre recombination occurs in target cells/tissues, causing deletion of a loxP site and the mT gene, leaving one loxP site in the DNA (1lox), and enabling the expression of the mG gene as opposed to other tissues in the body that contain two loxP sites (2lox). Histology and DNA analysis are performed in mouse tissues to confirm Cre recombination. Cell-free DNA, containing a mixture of 1lox and 2lox alleles released by various tissues through normal tissue turnover, can be extracted and analysed to reveal absolute contribution of cell-type/tissue of interest. [file 12920_2020_705_MOESM1_ESM.jpg]

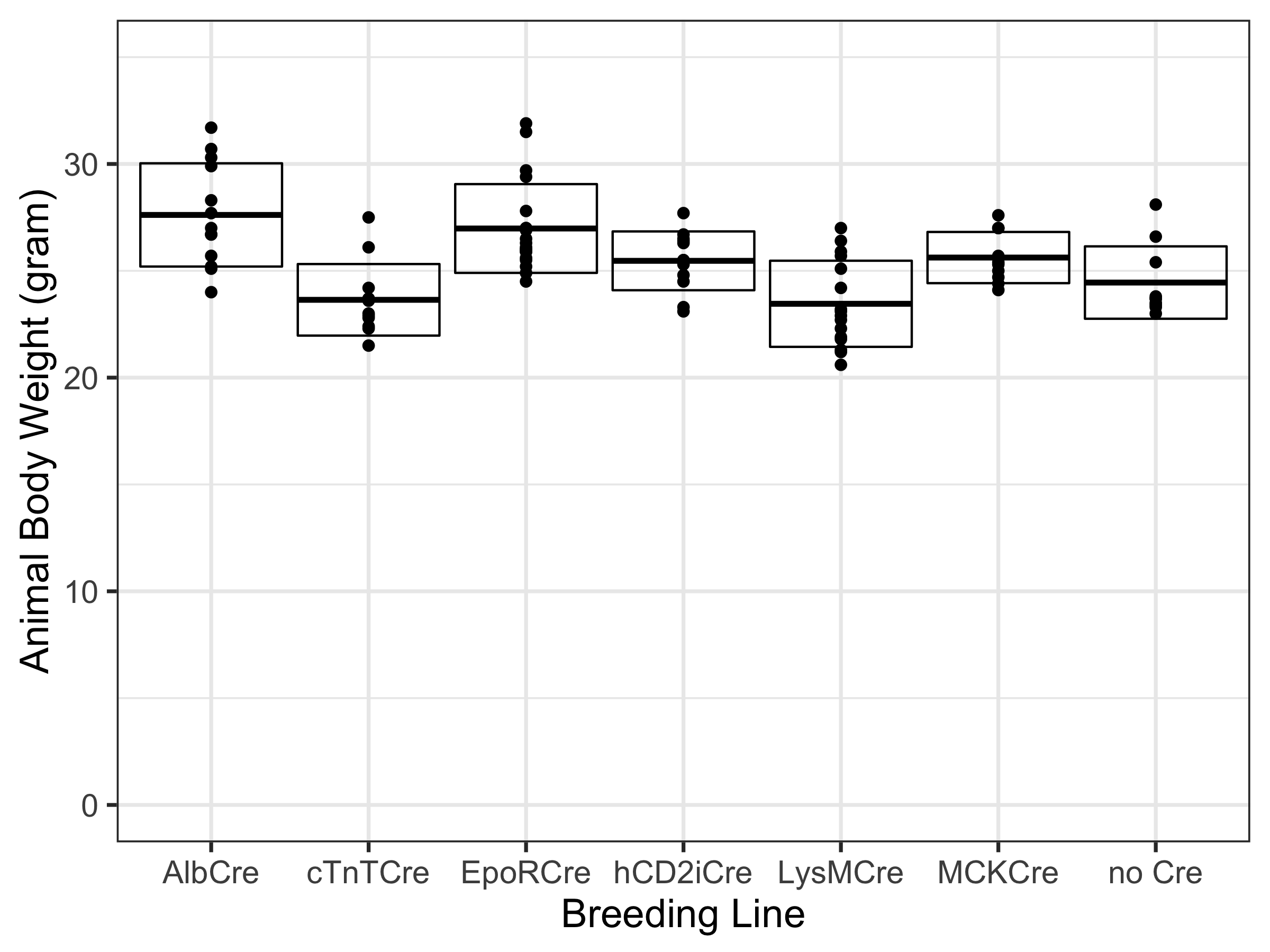

Supplement: Supplementary file 2 — Additional file 2: Figure S2. Body weight measurements from different mouse lines at 10–12 week old. Description of data: Body weight of mice (n > 10) ranges between 20.6 and 31.9 g. Tissue knockout mouse lines showed similar body weight to non-knockout control (no Cre). [file 12920_2020_705_MOESM2_ESM.tif]

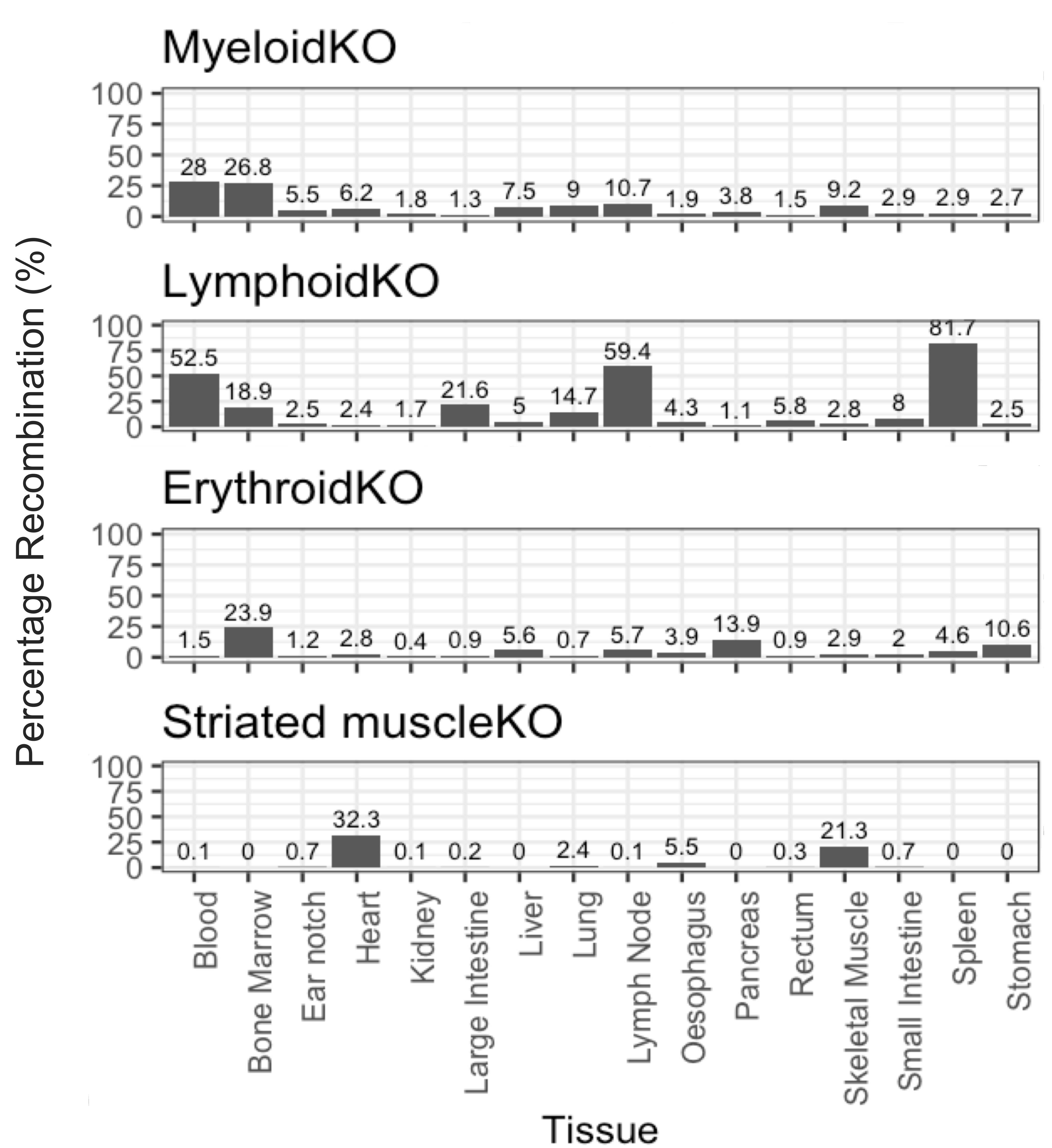

Supplement: Supplementary file 5 — Additional file 5: Figure S3. Specificity of Cre recombination across 16 mouse tissues in four knockout lines. Description of data: Knockout lines from top to bottom: myeloid, lymphoid, erythroid and striated muscle. [file 12920_2020_705_MOESM5_ESM.tif]

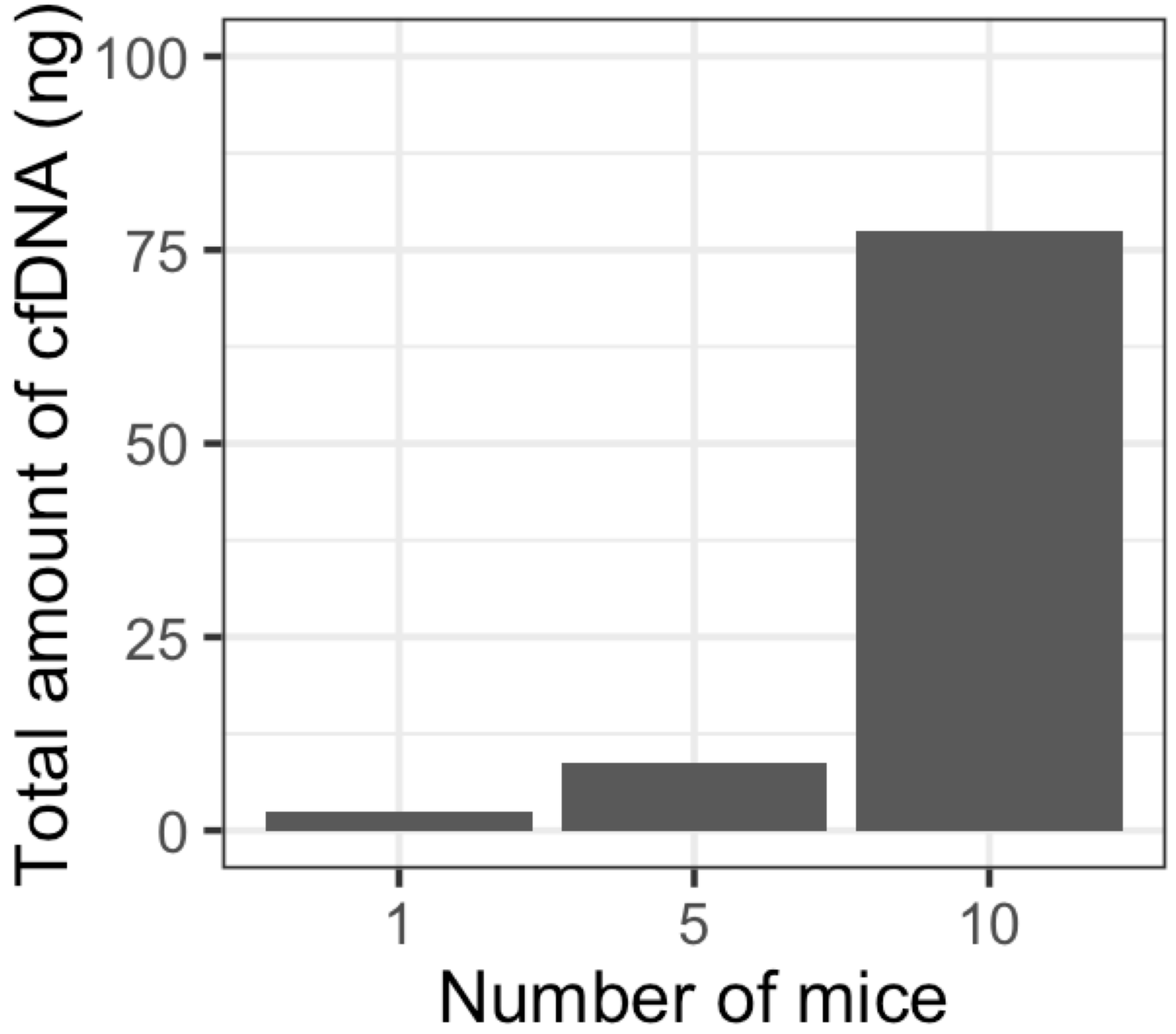

Supplement: Supplementary file 6 — Additional file 6: Figure S4. Total amount of cfDNA extracted from a single mouse, and pools of 5, and 10 C57BL/6 mice. Description of data: Total amount of cfDNA from a pool of 10 mice provided sufficient cfDNA input for at least duplicate measurements using ddPCR (20 ng for a single measurement of 1lox and 2lox alleles). [file 12920_2020_705_MOESM6_ESM.tif]

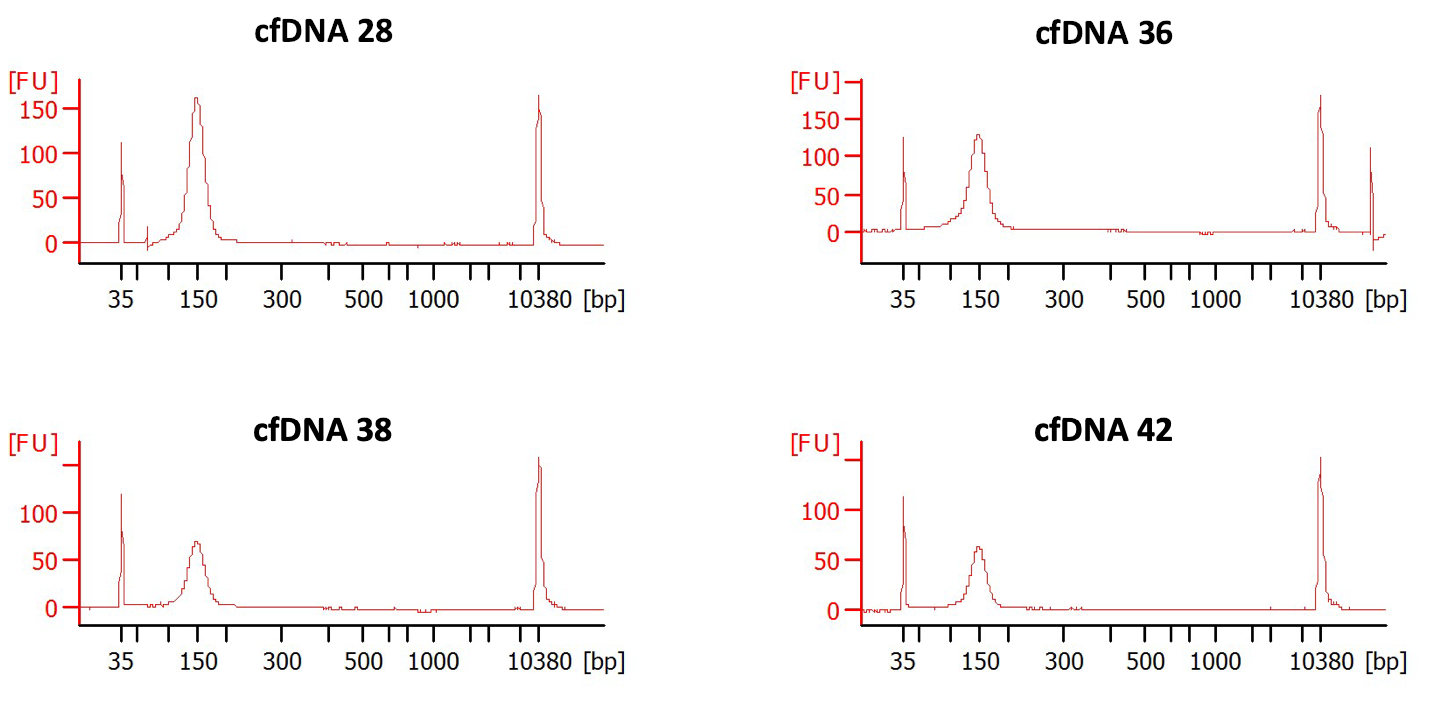

Supplement: Supplementary file 9 — Additional file 9: Figure S6. Fragment size of cfDNA following APAP overdose in four mice showed a majority contribution of mononucleosomal DNA (~ 150 bp). [file 12920_2020_705_MOESM9_ESM.tif]

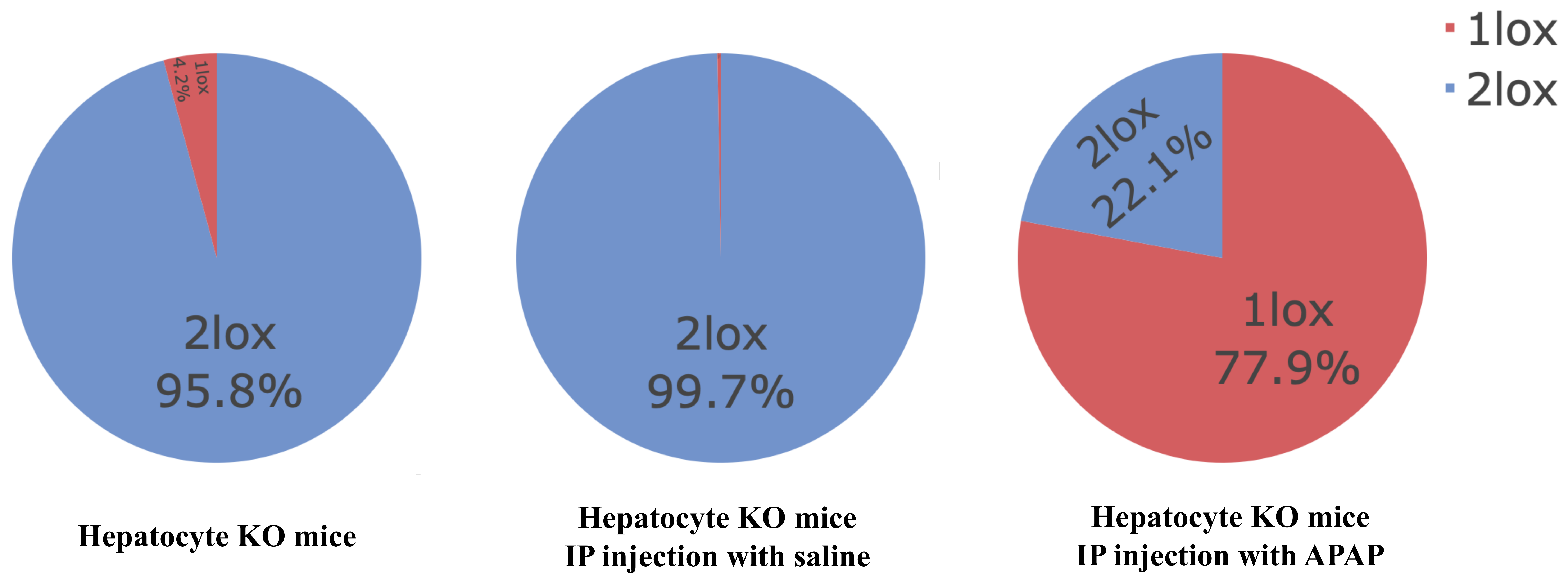

Supplement: Supplementary file 10 — Additional file 10: Figure S7. The effect of intraperitoneal injection on the tissue origins of cfDNA Description of data: The increase of hepatocyte contribution in the analysis of the tissue origins of cfDNA 8-h after APAP dosing in hepatocyte-specific knockout mice was not caused by intraperitoneal injection, as shown by a decrease of tissue contribution in mice injected with saline. [file 12920_2020_705_MOESM10_ESM.tif]
